# Field evaluation of two commercial feline immunodeficiency virus antibody detection point-of-care kits in domestic cats

**DOI:** 10.1186/s12917-026-05416-9

**Published:** 2026-04-14

**Authors:** Mahmoud S. Safwat, El shymaa A. Abdallah, Eman Beshry Abd-Elfatah, Ahmad Zaki Anwer, M. E. Ali, Eman A. Ahmed, Shimaa M. Abdullah, Dina A. Abdelkhalek, Omar S. Saeed, Reham Karam, Nehal M. Shahen, M. H. Ali, Saad A. Moussa, Elsayyad M. Ahmed, Rabab T. Hassanien, Ahmed F. Afify, Shimaa Nassef Abd-Elhafeiz, Samah Eid, Samah Elsayed M

**Affiliations:** 1 Department of Internal Medicine and Infectious Diseases (Infectious Diseases), Faculty of Veterinary Medicine, Cairo University, Giza, 12211 Egypt; 2 Educational Veterinary Hospital, Faculty of Veterinary Medicine, Zagazig University, Zagazig, 44511 Egypt; 3 Department of Animal Medicine, Infectious Diseases, Faculty of Veterinary Medicine, Zagazig University, Zagazig, 44511 Egypt; 4 Department of Internal Medicine and Infectious Diseases (Internal Medicine), Faculty of Veterinary Medicine, Cairo University, Giza, 12211 Egypt; 5 Department of Animal Medicine, Internal Medicine, Faculty of Veterinary Medicine, Suez Canal University, Ismailia, 41522 Egypt; 6 Department of Animal Medicine, Internal Medicine, Faculty of Veterinary Medicine, Zagazig University, Zagazig, 44511 Egypt; 7 Department of Virology, Faculty of Veterinary Medicine, Cairo University, Giza, 12211 Egypt; 8 Department of Virology, Faculty of Veterinary Medicine, Mansoura University, Mansoura, 35511 Egypt; 9 WEQAA Central Laboratory, National Centre for the Prevention and Control of Plant and Animal Diseases (WEQAA), Riyadh, 11454 Saudi Arabia; 10 Virology Research Department, Animal Health Research Institute (AHRI), Agriculture Research Center (ARC), Giza, 12618 Egypt; 11 Virology Research Department, Animal Health Research Institute (AHRI), Hurghada Branch, Agriculture Research Center (ARC), Hurghada, 84511 Egypt; 12 Department of Bacteriology, Animal Health Research Institute (AHRI). Agriculture Research Center (ARC), Giza, 12618 Egypt

**Keywords:** Cats, Diagnostic performance, Egypt, Immunochromatography, Rapid kits, Serological diagnosis

## Abstract

**Background:**

Timely diagnosis of feline immunodeficiency virus (FIV) is essential for informed clinical decision-making, particularly when screening cats before admission into multi-cat environments or assessing blood donor candidates. Point-of-care (PoC) antibody detection kits play a central role in these settings, but their clinical utility relies on independent evaluations under field conditions. This study aimed to assess the diagnostic performance of two commercially available PoC kits for which independent evaluation data are lacking.

**Methods:**

A total of 116 archived serum samples from a previous FIV survey in Egypt were included, comprising 62 FIV-infected and 54 FIV-non-infected cats. Infection status had been determined previously using a diagnostic panel including two PoC kits with demonstrated high accuracy and PCR. Sequencing of available positive PCR samples revealed the sole presence of a novel subtype “X-EGY.” All samples were tested in parallel using the two kits under evaluation and independently interpreted by three observers blinded to infection status. Diagnostic accuracy, sensitivity, and specificity were calculated. Positive predictive value (PPV) and negative predictive value (NPV) were estimated using the calculated kit sensitivity and specificity, and the local FIV prevalence. Additionally, inter-kit agreement was assessed using Cohen’s kappa statistic.

**Results:**

Both kits demonstrated 100% diagnostic specificity in the sample set used. However, substantial differences were observed in diagnostic sensitivity (98.39% versus 61.29%) and test line intensity among concordant positive results. In the context of high local FIV prevalence previously reported in Egypt (31.6%), both kits showed 100% PPV, whereas NPV differed markedly (99.26% versus 84.83%), reflecting differences in diagnostic sensitivity. The overall agreement between the kits was moderate (kappa = 0.58).

**Conclusion:**

This study demonstrates marked disparities in the diagnostic performance of two commercially available FIV PoC kits under identical local conditions. High-prevalence settings like Egypt require PoC kits with high sensitivity to ensure a reliable NPV and prevent missed infections. Additionally, the circulation of a divergent subtype among Egyptian cats can also impair detection, further necessitating the use of high-sensitivity kits. Together, these factors underscore the urgent need for evaluation of PoC kits under local epidemiological and virological conditions to inform evidence-based kit selection in practice.

**Supplementary Information:**

The online version contains supplementary material available at 10.1186/s12917-026-05416-9.

## Background

Feline immunodeficiency virus (FIV) is a well-recognized retrovirus affecting domestic cats worldwide [1]. It is an enveloped, single-stranded RNA virus of the genus *Lentivirus* within the family *Retroviridae* [2]. Due to its high mutation rate, FIV has evolved into seven established subtypes (A–F and U-NZ) since its discovery in 1987, with multiple inter-subtype recombinant strains continuing to emerge [3]. Recently, an eighth subtype, designated X-EGY, was identified in Egyptian cats, representing the only subtype detected across all sequenced samples in the first molecular characterization of FIV in Egypt [4].

FIV is fragile outside the host and requires prolonged, close contact for transmission, most commonly through deep bite wounds [2]. Transmission via blood transfusion is also possible, whereas mating or vertical transmission is considered rare [1, 5]. Consequently, FIV is more frequently detected in intact adult male cats and is of particular concern in shelters and other multi-cat environments [1].

The infection typically progresses through three stages: an acute phase, a prolonged subclinical (latent) phase, and a terminal phase [6]. The terminal phase is the most clinically significant, characterized by marked immunosuppression, including chronic or recurrent infections, atypical or opportunistic pathogens, and, less commonly, lymphoma. Neurological signs and myelosuppression may also occur [2].

Several strategies have been explored for controlling FIV, including vaccination and test-and-removal programs [1]. However, the high genetic diversity of FIV has made vaccine development particularly challenging. Only a single commercial vaccine (Fel-o-Vax^®^ FIV), containing subtypes A and D, was ever released, and it was subsequently withdrawn from most countries due to limited cross-subtype protection and vaccine-induced antibodies that interfered with serological testing for several years. At present, the vaccine remains available only in Japan, Australia, and New Zealand [7, 8].

Similarly, Control strategies relying on molecular screening are also limited. Polymerase chain reaction (PCR) assays often have reduced sensitivity and may produce false-negative results due to viral genetic diversity, particularly in regions where circulating subtypes are poorly characterized [4, 9, 10]. In addition, viral loads are typically low during the latent phase of infection, further reducing the likelihood of PCR detection [6].

In contrast, antibody-based serological detection typically is less affected by viral diversity [6]. Also, FIV-specific antibodies usually rise to detectably high levels within 1–2 months of infection and remain elevated until the clinical phase. Together, these make serology the primary and most reliable diagnostic tool for FIV, forming the foundation of control programs based on testing and removal [4, 6, 9]. Point-of-care (PoC) antibody detection kits are widely used in feline practice, enabling rapid, on-site identification of infected cats with minimal equipment requirements, ease of use, cost-effectiveness, and suitability for screening large numbers of cats in shelters [6]. Therefore, evaluating their performance and diagnostic accuracy is critically important.

While several PoC kits are commercially available, veterinary practitioners are advised to select those that have undergone independent field evaluation to ensure accurate results [6]. In this context, while some kits have been assessed in different cat populations and geographical regions [11–17], many remain unevaluated. Therefore, the present study aimed to provide the first independent, field-based evaluation of the diagnostic performance of two of these kits.

## Materials and methods

### Study design, source population, and serum samples

This study represents a field-based evaluation of two commercially available FIV antibody PoC kits using archived serum samples stored at − 80 °C. Serum samples originated from domestic cats previously examined during two epidemiological surveys investigating feline hemotropic mycoplasma infection (FHM) and FIV in three governorates (Cairo, Giza, and Al-Qalyubia), Egypt, between December 2022 and April 2024 [4, 18]. Only archived serum samples with sufficient volume obtained after completion of the original diagnostic investigations were eligible for inclusion in the current study. In this context, a total volume of ≥ 100 µL serum per sample was calculated as the minimum sufficient amount to perform testing with the two commercial kits under evaluation, including the manufacturers’ required input volumes and allowing for potential pipetting losses. Serum testing for the current study was conducted in June 2025, resulting in a variable storage period of 14–30 months. The current study was approved by the Institutional Animal Care and Use Committee at the Faculty of Veterinary Medicine, Cairo University (Vet CU 131020241041).

### FIV infection status

The FIV infection status of the included cats had been established independently of the present evaluation during the previous survey using a composite diagnostic approach [4]. This approach included two commercially available PoC kits, which simultaneously detect FIV antibodies and feline leukemia virus (FeLV) antigens (SNAP FIV Ab/FeLV Ag kit [IDEXX, USA] and Anigen FIV Ab/FeLV Ag kit [BioNote, South Korea]) together with a conventional PCR assay targeting the FIV *env*3–5 gene region. Both SNAP and Anigen PoC kits, as well as the PCR assay, were performed on whole blood samples. The SNAP FIV Ab/FeLV Ag kit is based on enzyme-linked immunosorbent assay (ELISA) technology, using the conserved matrix (P15) and capsid (P24) proteins as antigens, with reported diagnostic sensitivity and specificity ranging from 88.9 to 100% and 97–100%, respectively [13, 15–17]. The Anigen FIV Ab/FeLV Ag kit is based on immunochromatography technology and detects antibodies against the conserved transmembrane envelope protein (gp40), with reported sensitivity and specificity ranging from 88.9 to 100% and 99–100%, respectively [13, 15, 16].

Cats were classified as FIV-infected if they tested positive by at least two of the three diagnostic techniques, or if they were PCR-positive with sequence confirmation. FIV subtyping had been performed during the previous survey only for samples that tested positive by the env3–5 PCR assay.

### Health status and clinical classification

Comprehensive assessment of health status in FIV-infected cats, including history-taking, physical examination, routine hematological and biochemical analyses, and PCR for FeLV and FHM, was performed by clinicians specializing in veterinary internal medicine. Based on these findings, FIV-infected cats were classified into three groups: (i) clinically healthy cats, (ii) cats considered presumptively in the terminal stage if they displayed patterns of disease commonly associated with this stage, such as persistent or recurrent infections, opportunistic or atypical infections, lymphoma, or neurological signs [2], and (iii) cats showing clinical disease not typically associated with FIV. This classification was used to assess the potential impact of terminal-stage FIV infection on PoC kit sensitivity and the occurrence of false-negative results.

### Serological testing for FIV antibodies using the two commercial kits under evaluation

All archived serum samples were thawed at room temperature and tested in parallel for FIV antibodies using two commercial immunochromatography-based PoC kits routinely used in clinical practice in Egypt due to their availability and relatively lower cost. The first kit was Fassisi^®^ FeLFIV (Fassisi GmbH, Germany), which detects antibodies against the gp40 protein; the manufacturer reports a diagnostic sensitivity of 97.62% and a specificity of 98.54%. No publicly available data on how these manufacturer-reported performance estimates were generated, including the reference standard used, sample size, or the FIV subtypes evaluated; we contacted the manufacturer to request this information but did not receive any response.

The second kit was VDRG^®^ FeLV Ag/FIV Ab (Median Diagnostics). Following direct communication with the manufacturer, we were informed that this kit also utilizes the gp40 as the capture antigen. The manufacturer reported a diagnostic sensitivity of 96.8% and a specificity of 98.8%, based on internal validation against a commercial ELISA assay: FIV Ab ELISA 96 (Biopronix, Italy). Separate evaluations of VDRG kit across different FIV subtypes were not conducted, and the exact amino acid composition of the gp40 antigen was not disclosed.

For each PoC kit, a test result was considered valid only when the control line was present. All PoC kit results were independently and rigorously assessed by a panel of three authors blinded to the FIV infection status of the cats. Final interpretation was determined by consensus, defined as agreement of at least two panel members.

### Statistical analysis

The performance of both kits was evaluated by calculating diagnostic accuracy, sensitivity, and specificity according to the following equations [19]:

$$sensitivity\left(\%\right)=\frac{TP}{TP+FN}\times100$$

$$specificity\left(\%\right)=\frac{TN}{TN+FP}\times100$$

$$Accuracy\left(\%\right)=\frac{TP+TN}{n}\times100$$

Where:

TP (True positives): proportion of FIV-infected cats, as determined by the composite diagnostic panel, that tested positive by the PoC kit.

TN (True negatives): proportion of FIV-non-infected cats, as determined by the composite diagnostic panel, that tested negative by the PoC kit.

FP (false positives): proportion of FIV-non-infected cats that tested positive by the PoC kit.

FN (false negatives): the proportion of FIV-infected cats that tested negative by the PoC kit.

n: the total number of tested cats.

The positive predictive value (PPV) and negative predictive value (NPV) were calculated using the estimated sensitivity and specificity of the PoC kits and the previously reported prevalence of FIV in Egyptian cats, 31.6% [4], using the following formulas [19]:

$$positivepredictivevalue\frac{Sensitivity\times prevalence}{sensitivity\times prevalence+\left(1-specificity\right)\times(1-prevalence)}\times100$$

$$negativepredictivevalue\frac{specificity\times(1-prevalence)}{\left(1-sensitivity\right)\times prevalence+specificity\times(1-prevalence)}\times100$$

To assess agreement levels between the two PoC kits beyond chance, the Cohen’s Kappa (*κ*) statistic was calculated. The formula used was:

*κ* = $$\frac{Po-Pe}{1-Pe}$$

Where:

*Po:* The observed proportion of agreement.

*Pe: *The expected proportion of agreement by chance.

Interpretation of κ values followed the scale proposed by Landis and Koch [20]: 0.00–0.20 = slight, 0.21–0.40 = fair, 0.41–0.60 = moderate, 0.61–0.80 = substantial, and 0.81–1.00 = almost perfect agreement.

The 95% confidence intervals (CIs) were calculated as follows: exact Clopper–Pearson intervals were used for sensitivity, specificity, and accuracy, while standard logit confidence intervals were applied for predictive values. The asymptotic standard error method based on the normal approximation was used to calculate the 95% CI for κ.

All calculations were performed using MedCalc Software (Version 23.4.5; MedCalc Software Ltd., https://www.medcalc.org/en/calc/diagnostic_test.php; accessed December 10, 2025).

## Results

### Study population: demographics, FIV infection status, and clinical health

A total of 116 cats with archived leftover serum samples were included in the study. Most were shelter-housed (*n* = 103), with the remainder being client-owned (*n* = 13). All were mixed-breed cats, classified by age into kittens (< 1 year; *n* = 5), young adults (1–6 years; *n* = 45), mature adults (7–10 years; *n* = 52), and seniors (> 10 years; *n* = 14). None of the cats in the kitten group was younger than 6 months of age. The population comprised 50 females and 66 males; all females but one were sexually intact, whereas most males (*n* = 48) were castrated. No FIV vaccination is practiced in Egypt, and none of the cats in the original survey originated from countries where the FIV vaccine is used; therefore, all cats included in the current study were considered unvaccinated against FIV.

Based on the composite diagnostic panel, 62 cats were classified as FIV-infected and 54 as FIV-non-infected. Of the 62 FIV-infected cats, 8 tested positive by all three assays (PCR, SNAP^®^, and Anigen^®^), whereas 54 tested positive by both Anigen^®^ and SNAP^®^ only. All FIV-non-infected cats tested negative across all three assays. Individual animal results for the composite diagnostic panel and the two commercial PoC kits evaluated in this study are provided in Supplementary Table 1. FIV subtyping of the eight PCR-positive cats revealed that all were infected with the novel X-EGY subtype.

Among the 62 FIV-infected cats, 32 were clinically healthy, while 30 were classified as diseased. Of the diseased cats, 25 were considered presumptively in the terminal stage of FIV infection, exhibiting clinical conditions associated with immunosuppression commonly reported in this stage, including chronic or recurrent upper respiratory infection in 19 cats; FHM-associated anemia caused by *Candidatus* Mycoplasma haematominutum or *Candidatus* Mycoplasma turicensis in four cats; combined chronic/recurrent upper respiratory infection with FHM-associated anemia caused by *Candidatus* Mycoplasma haematominutum- or *Candidatus* Mycoplasma turicensis in one cat; and pancytopenia in one cat. The remaining five cats exhibited conditions not typically associated with FIV, including chronic renal failure (*n* = 2), diabetes mellitus (*n* = 1), hypothyroidism (*n* = 1), and feline aortic thromboembolism (*n* = 1).

### Performance of the two commercial FIV antibody detection kits under evaluation

None of the kits tested across both brands produced invalid results. Complete consensus among the three blinded observers was achieved for all Fassisi^®^ kit results, whereas it was not reached for 10 VDRG^®^ kit results from FIV-infected cats; in these cases, two observers interpreted the result as negative.

Fassisi^®^ correctly identified 61 of 62 FIV-infected cats, yielding a diagnostic sensitivity of 98.39% (95% CI: 91.34–99.96%). In contrast, VDRG^®^ detected only 38 of 62 infected cats, corresponding to a sensitivity of 61.29% (95% CI: 48.07–73.40%) (Table 1). Neither kit produced false-positive results among FIV-non-infected cats, resulting in a specificity of 100% for both (95% CI: 93.40–100.00%).

**Table 1:** The diagnostic performance of Fassisi^®^ and VDRG^®^ kits in feline immunodeficiency virus (FIV) infection

		FIV infection statusᵃ		
		FIV-infected	FIV-non-infected	
Fassisi^®^ kit	Positive	61ᵇ	0 ͩ	61
	Negative	1ͨ	54ͤ	55
VDRG^®^ kit	Positive	38ᵇ	0 ͩ	38
	Negative	24ͨ	54ͤ	78
		62	54	*n* = 116

ᵃ The FIV infection status was determined by a composite diagnostic panel, consisting of two rapid antibody detection kits (SNAP^®^ and Anigen^®^) and one conventional PCR targeting the *env*3-5 gene

ᵇ True positives (TP)

ͨ False negatives (FN)

ͩ False positives (FP)

ͤ True negatives (TN)

The diagnostic performance of each kit brand was determined based on the following values:

$$sensitivity\left( \% \right)=\frac{TP}{TP+FN}\times100;$$

for Fassisi^®^, sensitivity = $\frac{61}{61+1}\times100=$ 98.39%

for VDRG^®^, sensitivity = $\frac{38}{38+24}\times100=$ 61.29%

$$Specificity\left(\%\right)=\frac{TN}{TN+FP}\times100;$$

for both Fassisi^®^ and VDRG^®^, specificity = $\frac{54}{54+0}\times100=$ 100.00%

$$Accuracy\left(\%\right)=\frac{TP+TN}{n}\times100;$$

for Fassisi^®^, Accuracy = $\frac{61+54}{116}\times100=$ 99.14%

for VDRG^®^, Accuracy = $\frac{38+54}{116}\times100=$ 79.31%

Based on these values, Fassisi^®^ demonstrated a diagnostic accuracy of 99.14% (95% CI: 95.34–99.91%), whereas VDRG^®^ showed an accuracy of 79.31% (95% CI: 71.12–85.69%).

Using the previously reported FIV prevalence in Egyptian cats (31.6%), the PPV of both kits was 100.00%, with lower 95% CI bounds of 94.13% for Fassisi^®^ and 90.75% for VDRG^®^. The NPV was 99.26% for Fassisi^®^ (95% CI: 95.05–99.89%) and 84.83% for VDRG^®^ (95% CI: 80.35–88.44%).

**Fig. 1:**
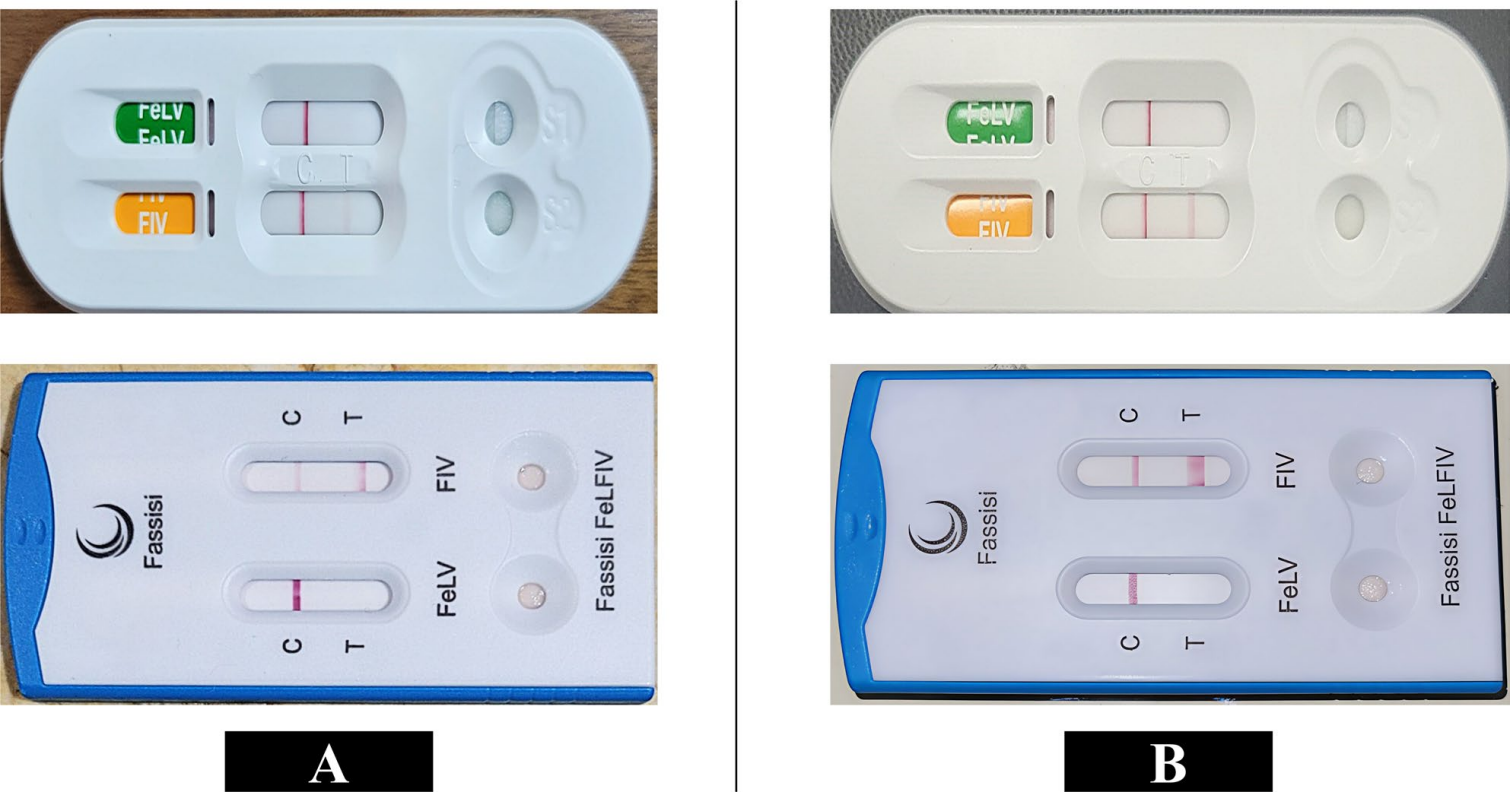
Comparison of FIV point-of-care kit test line intensity in two representative cats. For each panel, VDRG^®^ kits (top) consistently showed weaker positive test lines compared with Fassisi^®^ kits (bottom) when testing the same cat. Panel A (cat no. 218) illustrates the predominant VDRG^®^ result pattern (36/38 positive samples) characterized by weak test lines, whereas Panel B (cat no. 104) represents one of the two VDRG^®^-positive cases with a more distinct test line. In both panels, Fassisi kits exhibit visibly stronger test lines

Both kit brands yielded concordant results for 91 of the 116 samples (37 positive and 54 negative). Discordant results were observed in 25 samples: 24 were positive by Fassisi^®^ but negative by VDRG^®^, while one sample was positive by VDRG^®^ but negative by Fassisi^®^. Overall, the two kits demonstrated moderate agreement (κ = 0.58; 95% CI: 0.44–0.71) (Table 2). Among the 37 samples that tested positive by both PoC kits, Fassisi^®^ consistently produced a stronger test-line intensity than VDRG^®^, as illustrated in (Fig. 1).

**Table 2:** Strength of agreement between Fassisi^®^ and VDRG^®^ kits for the detection of feline immunodeficiency virus (FIV) infection

		Fassisi^®^		
		Positives	Negatives	
VDRG^®^	Positives	37	1	38
	Negatives	24	54	78
		61	55	*n* = 116

κ = 0.58

## Discussion

This study provides novel data regarding the diagnostic performance of two FIV antibody detection PoC kits in Egyptian cats. Three methodological criteria should be considered in studies evaluating diagnostic techniques, including: (i) statistically calculated sample size and random sampling, (ii) blinded interpretation of test results, and (iii) comparison to a reference standard [12, 13, 15].

Sample size and random sampling, the first criterion, could not be fully achieved in the present study, as it relied on archived leftover serum from previous surveys. While convenient, using only remaining samples may yield a subset that does not fully represent the source population. In the present study, this approach resulted in a sample with a higher proportion of FIV-infected cats than expected in the general population, producing an FIV-enriched composition. Although this enrichment is not ideal for directly estimating population-dependent metrics such as PPV and NPV, it increases precision for sensitivity, specificity, and accuracy calculations, enhancing the statistical power for comparing the two kits [13]. PPV and NPV were therefore adjusted using the known prevalence of FIV in Egyptian cats to provide realistic estimates for field application.

Blinded interpretation of PoC kit results, the second criterion, was met by having multiple observers independently assess each PoC kit, with consensus used for any ambiguous results. This approach minimizes subjective bias and aligns with best practices for diagnostic evaluations [12, 13, 15].

Comparison to a reference standard, the third criterion, ideally involves Western blot (WB), which is widely recognized as the gold standard for FIV antibody detection [9, 15]. WB is not available in Egypt, as it is neither produced locally nor imported, and the resources required for in-house WB development, including specific pathogen-free cats and established feline lymphoid cell lines (e.g., MYA-1 or FL4) for viral isolation and antigen preparation, are also unavailable. Consequently, WB is technically unfeasible under local conditions and represents a limitation to this study.

In the absence of WB, the original survey employed a composite diagnostic panel combining PCR and two PoC kits, with a cat classified as FIV-infected if two of the three assays were positive [4]. Using a composite diagnostic panel as an alternative gold standard was described in previous studies [11, 16]. While the PCR assay adopted in our panel showed very low sensitivity (13.1%), possibly due to the high genetic divergence of the local FIV subtype from the subtypes A and B for which the assay was originally designed [21, 22], the two PoC kits demonstrated complete agreement in all samples.

This agreement provides a reliable indication of infection and is supported by several factors: (i) These PoC kits target different viral proteins (SNAP^®^: p15/p24; Anigen^®^: gp40) [8], achieving multi-antigen detection analogous to WB and effectively fulfilling the WB requirement for reactivity to at least two viral proteins [15, 23, 24]. SNAP^®^ also includes a negative control to minimize nonspecific binding. (ii) Both kits have been validated in independent studies across multiple FIV variants and showed high sensitivity using virus isolation or composite panels as references, indicating they detect conserved epitopes largely unaffected by viral variation [13, 16, 17]. (iii) Sequential testing with two PoC kits is recommended in international guidelines: a positive result on the first kit is confirmed with a second from another manufacturer, with concordant positives defining infection [1, 6, 8]. While dual PoC kit agreement does not replace WB as a laboratory gold standard, it represents the highest level of diagnostic certainty achievable in routine clinical settings where WB is unavailable.

Both evaluated kits showed no false-positive results, demonstrating 100% specificity. False-positive FIV serological reactions are most commonly associated with maternally derived antibodies in kittens younger than six months or with vaccine-induced antibodies [6]; neither condition applied in the present study, as all cats were adults and none had been vaccinated. Because FIV vaccination is not practiced in Egypt and infection predominantly affects adult cats, this limitation is unlikely to affect the local applicability of our findings, although caution is warranted when interpreting results in cats imported from regions where vaccination is routinely used.

Furthermore, Egypt is reported to have one of the highest endemic FIV prevalence rates worldwide [4, 25, 26], which inherently increases the positive predictive value of serological tests [13]. This epidemiological context further supports the clinical reliability of positive PoC kit results, particularly in high-risk settings such as animal shelters [13].

While the Fassisi^®^ kit demonstrated a high diagnostic sensitivity (98.39%), the VDRG^®^ kit yielded a substantially lower sensitivity (61.29%) due to a high number of false-negative results. This difference is particularly consequential under high-prevalence conditions like Egypt because the reliability of a negative PoC kit result, reflected by the NPV, is strongly dependent on its sensitivity; consequently, selecting PoC kits with high sensitivity is essential to minimize undetected infections and to support effective FIV control strategies [13, 27].

One potential explanation for the low sensitivity of VDRG^®^ is the high genetic diversity of the novel FIV variant circulating in Egyptian cats. Although serological testing is generally less affected by antigenic variation, this is not absolute, and some laboratory-based or PoC kits may still be influenced if the antigens they use are not well conserved. Indeed, a recent study reported false-negative results in a laboratory-based TM-ELISA, potentially due to imported antigenic variation [28]. It is therefore possible that Anigen^®^ and SNAP^®^ in our previous study, and Fassisi^®^ in the present study, which demonstrated high sensitivity, incorporate antigens well conserved in the Egyptian variant, whereas the VDRG^®^ may rely on targets that are less well matched, resulting in reduced reactivity.

Although VDRG^®^ incorporates gp40 as its target antigen, similar to the Anigen^®^ and Fassisi^®^, this does not refute antigenic variation as a possible cause, as gp40 is a large envelope protein, and early epitope-mapping studies demonstrated that distinct gp40-derived peptides vary substantially in their immunoreactivity and diagnostic sensitivity [29]. Accordingly, labeling different commercial PoC kits as “gp40-based” does not imply that identical epitopes or peptide sequences are used across kits. Supporting this, Westman et al. reported that Witness^®^ employs a defined 14-amino-acid gp40 peptide, whereas the exact gp40 epitope composition of other commercial kits, including Anigen^®^, is not publicly disclosed [7]. As the exact amino acid sequences of the antigens in these commercial PoC kits are not publicly available, and no comparative studies have assessed the performance of Fassisi^®^ and VDRG^®^ across multiple FIV subtypes, this explanation remains speculative.

Another contributing factor may be the lower analytical sensitivity of VDRG^®^ compared with Fassisi^®^. This lower sensitivity could result in false-negative results when antibody concentrations are near the detection limit. This interpretation is supported by the consistently weaker test-line intensity observed with VDRG^®^ compared to Fassisi^®^ in concordant positive samples (Fig. 1). As serial dilution of antibody titers was not performed, this explanation should be framed as a hypothesis.

FIV infection stage may further compound this issue: approximately half of the FIV-infected cats were presumptively classified as being in the terminal stage of infection, a phase characterized by functional immunodeficiency and potentially reduced antibody titers. Although titers generally remain above the detection threshold of most commercial PoC kits [6], a combination of reduced antibody levels and lower analytical sensitivity may contribute to the false-negative results observed with VDRG^®^. However, because objective immunological and virological markers (e.g., CD4 count, CD4:CD8 ratio, viral loads) to define disease stage were not available and the study design was cross-sectional rather than longitudinal, infection stage could not be defined objectively. Consequently, this interpretation should be regarded as exploratory rather than definitive.

In addition, although both kits are approved by the manufacturers for use with either whole blood or serum, matrix-related effects cannot be entirely excluded. The use of archived serum rather than whole blood may have contributed to reduced performance in VDRG^®^, whereas the preserved high sensitivity of Fassisi^®^ suggests that any such effect was likely kit-specific rather than universal. As no direct comparative evaluation between serum and whole blood was conducted for either kit in this study, this explanation remains speculative.

Taken together, the observed false-negative results with VDRG^®^ may reflect a combination of factors, including antigenic variation, lower analytical sensitivity, potentially reduced antibody titers in cats with advanced-stage infection, and possible matrix-related effects associated with the use of archived serum samples. Since this study was designed to document diagnostic discordance between commercially available PoC kits under field conditions, mechanistic explanations are, therefore, presented as hypotheses for future targeted investigations, rather than as established causality. Additionally, where available, WB would be valuable to further explore the potential impact of antigenic variation.

Beyond the aforementioned constraints regarding sample size, random sampling, and the unavailability of a WB reference standard, this study has additional limitations that warrant consideration. First, this retrospective analysis was predominantly composed of shelter-housed cats, with fewer client-owned cats represented. Although the FIV prevalence used to calculate NPV and PPV reflects the combined population of both shelter- and client-owned cats [4], the overrepresentation of shelter-housed cats, coupled with their significantly higher FIV prevalence compared to client-owned cats, requires caution when generalizing these findings to the broader feline population in Egypt. However, since most client-owned cats are strictly indoor and have minimal exposure risk, this sample reflects the population most likely to be tested with PoC kits in practice.

Second, whole blood was not evaluated, despite being the most commonly used matrix in routine clinical practice [11, 15]. This reflects the retrospective nature of the study and manufacturer instructions indicating that, while whole blood or serum may be used for immediate testing, whole blood should not be frozen for delayed analysis, whereas serum can be frozen and stored. Given the documented stability of serum IgG at ≤ − 20 °C and the minimal impact of freeze–thaw cycles on antibody integrity and serologic performance across a wide range of pathogens [30], serum was considered the most suitable choice for a retrospective study of this nature.

Future studies incorporating larger, randomly sampled populations, fresh whole blood, and broader FIV subtype diversity are warranted to confirm these results and improve the applicability of the findings.

## Conclusion

PoC kits are essential for timely FIV diagnosis and informed clinical decision-making. In this field-based evaluation, two antibody detection PoC kits showed notable differences in diagnostic performance in Egyptian cats, where FIV prevalence is high, and a highly divergent local subtype circulates. One kit demonstrated high sensitivity, whereas the other showed lower sensitivity; additionally, a marked difference in test line intensity between the two kits was observed, reflecting the differences in diagnostic sensitivity. In the context of marked viral diversity and high prevalence, selecting PoC kits with high sensitivity, and therefore high NPV, is critical to minimize missed infections and support appropriate management of FIV-positive cats.

These findings emphasize that the performance of commercially available PoC kits cannot be assumed to be universal and highlight the importance of independent field evaluations tailored to local epidemiological and virological conditions. Further studies using different sample matrices, broader subtype diversity, and vaccinated populations are warranted to enhance the generalizability of these results.

## Supplementary Information

Supplementary Material 1.

## Data Availability

All data generated and analyzed in this study were reported in the manuscript.
